# Regulation of mild cognitive impairment associated with liver disease by humoral factors derived from the gastrointestinal tract and MRI research progress: a literature review

**DOI:** 10.3389/fnins.2023.1206417

**Published:** 2023-06-16

**Authors:** Tianning Sun, Maohui Feng, Anne Manyande, Hongbing Xiang, Jun Xiong, Zhigang He

**Affiliations:** ^1^Department of Anesthesiology, Hubei Key Laboratory of Geriatric Anesthesia and Perioperative Brain Health, Wuhan Clinical Research Center for Geriatric Anesthesia, Tongji Hospital, Tongji Medical College, Huazhong University of Science and Technology, Wuhan, Hubei, China; ^2^Department of Gastrointestinal Surgery, Wuhan Peritoneal Cancer Clinical Medical Research Center, Zhongnan Hospital of Wuhan University, Hubei Key Laboratory of Tumor Biological Behaviors, Hubei Cancer Clinical Study Center, Wuhan, Hubei, China; ^3^School of Human and Social Sciences, University of West London, London, United Kingdom; ^4^Center for Liver Transplantation, Union Hospital, Tongji Medical College, Huazhong University of Science and Technology, Wuhan, China

**Keywords:** cognitive impairment, liver disease, humoral factor, MRI research, brain

## Abstract

Patients with liver disease are prone to various cognitive impairments. It is undeniable that cognitive impairment is often regulated by both the nervous system and the immune system. In this review our research focused on the regulation of mild cognitive impairment associated with liver disease by humoral factors derived from the gastrointestinal tract, and revealed that its mechanisms may be involved with hyperammonemia, neuroinflammation, brain energy and neurotransmitter metabolic disorders, and liver-derived factors. In addition, we share the emerging research progress in magnetic resonance imaging techniques of the brain during mild cognitive impairment associated with liver disease, in order to provide ideas for the prevention and treatment of mild cognitive impairment in liver disease.

## Introduction

Liver disease patients are prone to various cognitive impairments (Sun et al., 2022; Yang et al., 2022; He et al., 2023). Even in healthy populations, plasma transaminase levels are significantly negatively correlated with memory ability (Kamada et al., 2016). Hepatic encephalopathy (HE) is a brain dysfunction caused by acute or chronic liver dysfunction and/or portosystemic shunts, mainly characterized by neurological and psychiatric abnormalities, cognitive and motor function changes (Rose et al., 2020). According to the severity of the patient’s condition, HE can be divided into minimal hepatic encephalopathy (MHE) and overt hepatic encephalopathy (OHE) (Bajaj et al., 2011). MHE occurs during the early stage of HE and is commonly seen in patients with chronic liver cirrhosis. These patients do not have clinical symptoms, but there are cognitive changes indicated by neuropsychological testing (Moran et al., 2021). It has been reported that the incidence of MHE in patients with liver cirrhosis can be as high as 20–80% (Lauridsen et al., 2011; Allampati et al., 2016). MHE seriously affects patients’ work and quality of life, but it especially increases the risk of traffic accidents (Bajaj et al., 2009; Mina et al., 2014). In addition, MHE is prone to progress to OHE (Hartmann et al., 2000), increasing the risk of hospitalization and death of patients (Kim et al., 2019).

As well as MHE, liver diseases such as non-alcoholic fatty liver disease (NAFLD) (Kjaergaard et al., 2021), chronic hepatitis C (Mazzaro et al., 2021), cholestatic liver disease (Phaw et al., 2021), and liver transplantation (Getsuwan et al., 2021) also have mild cognitive impairment. Although current research cannot classify them as MHE, mild cognitive impairment in these liver diseases may be a precursor of OHE and is associated with poor prognosis in patients (Collie, 2005). Once cognitive impairment progresses to OHE, the patient’s mortality rate will greatly increase (Yanny et al., 2019), and permanent central nervous system damage may occur. Even with liver transplantation (LT) treatment, patients will not completely recover cognitive function (Kornerup et al., 2019). Therefore, early understanding of the mechanism of mild cognitive impairment in liver disease is crucial for improving patient quality of life and prognosis by preventing the progression of HE. This review focuses on the changes in the brain during mild cognitive impairment related to liver disease, in order to better understand the mechanism of cognitive impairment and provides new insights for the prevention and treatment of mild cognitive impairment related to liver disease.

## Liver disease-related mild cognitive impairment status

In 1970, Zeegen et al. (1970) first reported that 47% of patients undergoing portal decompression surgery experienced brain dysfunction. Subsequently, up until 2011, there has been a gradual increase in research of mild cognitive impairment in chronic liver disease, when Bajaj et al. (2011) described cognitive impairment that appears as MHE before HE symptoms in patients with cirrhosis. In China, the main cause of cirrhosis is viral hepatitis, and the incidence of MHE among hospitalized patients with cirrhosis can reach up to 40% (Xu et al., 2019). It has been reported that cognitive impairment exists in chronic hepatitis C (HCV) patients even before the appearance of cirrhosis (Ibáñez-Samaniego et al., 2022), with more than 50% of HCV patients experiencing cognitive impairment (Mazzaro et al., 2021), antiviral therapy can significantly improve patients’ cognitive function by eradicating HCV (Kraus et al., 2013; Vaghi et al., 2020).

NAFLD is known to be the leading cause of chronic liver disease worldwide, affecting a quarter of the global population (Younossi et al., 2019). Cognitive impairment is also a common extrahepatic complication of NAFLD. According to investigations, up to 70% of NAFLD patients have memory loss and forgetfulness (Kjaergaard et al., 2021). A recent systematic review of 11 observational studies showed that NAFLD patients have cognitive impairments in multiple aspects such as “general cognition” and “mental speed and attention.” An et al. (2019) also found that cognitive impairment in NAFLD patients was related to elevated plasma liver enzymes and abnormal blood lipids. Moreover, Liu Q. et al. (2021) showed that the incidence of cognitive impairment in middle-aged and elderly NAFLD patients was significantly higher than in non-NAFLD patients in a longitudinal cohort study.

In cholestatic liver diseases, primary biliary cholangitis (PBC) patients often have symptoms of cognitive impairment, mainly manifested as memory loss, attention deficit, and psychomotor dysfunction. Newton et al. (2008) ascertained that about 53% of PBC patients have attention or memory impairment, and the severity of cognitive impairment is not related to the severity of the liver disease, such as biochemical indicators and histological damage, indicating that this cognitive impairment is largely unrelated to HE. Liu Y. et al. (2021) also reported that the health-related quality of life of Chinese PBC patients is impaired, and influenced by factors such as gender and age, with women and elderly patients more prone to cognitive impairment.

Liver transplantation (LT) is the ultimate treatment for end-stage liver disease. Previous studies determined that HE is completely reversible after LT, but increasing evidence suggests that patients still have varying degrees of cognitive impairment after the liver function has been restored by transplantation (Teperman and Peyregne, 2010; Kornerup et al., 2019). It has been reported that up to 30% of patients still have neurological sequelae after LT (Weiss and Thabut, 2019), and since the diseased liver has been replaced by a healthy liver, this post-LT cognitive impairment cannot yet be defined as HE. Furthermore, Campagna et al. (2014) showed that post-LT cognitive impairment is influenced by pre-transplant brain disease. Patients with obvious HE before transplantation had poorer overall cognitive function after LT than those without obvious HE, suggesting that post-LT cognitive impairment may be related to permanent brain damage caused by OHE to the central nervous system (Garcia-Martinez et al., 2011). Besides, hepatic ischemia–reperfusion injury (I/R) occurring during the perioperative period also has adverse effects on cognition (He et al., 2023). Many basic research studies have reported cognitive impairments in experimental animals after experiencing hepatic I/R injury (Wu et al., 2019; Wang et al., 2022).

In summary, mild cognitive impairment is very common in liver diseases. Patients not only suffer from impaired personality and activities of daily living ability but also experience a severe decline in their quality of life. Moreover, it creates a significant burden of care and psychological impact on their families (Fabrellas et al., 2020; Shrestha et al., 2020). Although the etiology and clinical manifestations of liver disease-related mild cognitive impairment are not entirely the same, their underlying mechanisms are very similar. The following section summarizes the brain changes in liver disease-related mild cognitive impairment.

### The mechanism underlying mild cognitive impairment in liver disease

#### Hyperammonemia

Ammonia is a neurotoxic substance primarily produced in the intestine by the metabolism of proteins and amino acids followed by intestinal bacterial degradation. Dysbiosis of the gut microbiota has been shown to increase ammonia production (Kang et al., 2016). Some preclinical studies have demonstrated that dysbiosis is associated with cognitive impairment and hyperammonemia in HE rats (Higarza et al., 2019). Since the elimination of ammonia depends on the liver’s ability to convert ammonia, impairment of liver function due to the lack of glutamine synthetase in the liver can result in the accumulation of ammonia in the body and cognitive changes (Qvartskhava et al., 2015). Elevated blood ammonia can freely pass through the blood–brain barrier, causing inflammation of the nervous system and leading to cognitive impairment (Bobermin et al., 2020; Jaffe et al., 2020)， however, treating HE symptoms can be achieved by reducing central ammonia levels and improving inflammation (Jayakumar et al., 2015; Lu et al., 2020). Ammonia is detoxified by converting it into glutamine by astrocytes in the central nervous system (Jayakumar and Norenberg, 2016), but glutamine has permeability, and its accumulation can cause swelling and functional impairment of astrocytes (Rama Rao and Norenberg, 2014) and brain edema (Cudalbu and Taylor-Robinson, 2019). Although high blood ammonia levels are considered a core factor in causing HE (Aldridge et al., 2015; Hadjihambi et al., 2018), their role in mild cognitive impairment associated with liver disease is not significant and is only observed in MHE patients with increased blood ammonia concentration levels and decreased brain ammonia metabolism (Lockwood et al., 1991). In addition, Higarza et al. (2019) showed that cognitive impairment in NAFLD rats was related to increased blood ammonia levels, but only mild ammonia elevation was observed in NAFLD patients (Felipo et al., 2012).

#### Neuroinflammation

Neuroinflammation is a common feature of various liver disease-related mild cognitive impairments. The immune cells of the central nervous system are microglia (Sheeler et al., 2020), which can monitor synaptic activity, pathogens, and injuries in the local environment and have phagocytic and synaptic remodeling-promoting functions. Microglial activation is a significant feature of neuroinflammation and is accompanied by an increase in cytokines and chemokines (Sheeler et al., 2020; Woodburn et al., 2021). It has been reported that MHE patients have significantly higher levels of inflammation, such as IL-6 and C-reactive protein, compared to normal individuals (Shawcross et al., 2007). Nonetheless, Shawcross et al. (2004) found that hyperammonemia in cirrhotic patients could only induce cognitive impairment when an inflammatory response occurred. Patients did not have cognitive impairment after the inflammation subsided, indicating that inflammation plays an important role in cognitive impairment in MHE patients. Neuroinflammation is also an important cause of cognitive impairment in NAFLD patients (Kjaergaard et al., 2021). Balzano et al. (2018) showed that NASH patients had neuroinflammatory manifestations such as neuronal loss, microglial and astrocytic activation, and lymphocyte infiltration in the cerebellum. Animal experiments have indicated that the neurological dysfunction of NAFLD is related to neuroinflammation (Veniaminova et al., 2020). Similarly, Bokemeyer et al. (2011) and Pflugrad et al. (2016) observed microglial and astrocytic activation and other neuroinflammatory manifestations in HCV patients. Although there is no evidence of cholestatic liver disease-associated neuropathy, it has been reported that NF-KB is activated in cholestatic liver disease, and NF-KB is associated with neuropathic inflammation and cognitive impairment (Phaw et al., 2021).

#### Brain energy metabolism disorders

The brain has a high energy demand, consuming 20% of the body’s energy despite accounting for only 2% of its weight (Magistretti and Allaman, 2018). Glucose is the brain’s primary source of energy, providing over 95% of its energy needs through the tricarboxylic acid (TCA) cycle in neurons under physiological conditions (Bordone et al., 2019). However, the astrocyte-neuron lactate shuttle hypothesis (Magistretti and Allaman, 2018) suggests that glucose is mainly metabolized to lactate in astrocytes. When neuronal activity increases, the released glutamate can trigger astrocytes to uptake glucose and produce lactate as a supplemental energy source for neurons (Pellerin et al., 2007; Huang et al., 2019).

Elevated brain lactate levels in hepatic encephalopathy (HE) patients are typically regarded as a sign of cerebral energy failure (Bosoi and Rose, 2014). The accumulation of lactate in brain cells can cause cell swelling and brain edema, and interfere with communication, metabolism, and neurotransmission between astrocytes and neurons (Bosoi and Rose, 2014). Jiménez et al. (2010) compared the serum 1H-NMR metabolome of healthy controls (*n* = 69), cirrhotic patients without minimal hepatic encephalopathy (MHE) (*n* = 62), and cirrhotic patients with MHE (*n* = 39), and found that MHE patients had increased glucose and lactate concentration levels. Basic research also suggests that bile duct ligation (BDL) induced HE in rats which results in significant lactate accumulation in the cerebellum, hippocampus, and striatum (Simicic et al., 2019). Additionally, Hadjihambi et al. (2017) demonstrated that hyperammonemia induced by BDL in HE rats can impair the function of lactate transporters and astrocyte-neuron lactate shuttle, leading to neuronal energy metabolism disorders.

Apart from metabolic substrates, adequate oxygen supply is needed for the normal functioning of the brain. The energy metabolism of the brain is regulated by the neurovascular unit (NVU), which consists of neurons, glial cells, and vascular cells (Ahmad et al., 2020). Astrocytes strictly regulate cerebral blood flow through the NVU, thereby regulating the nutrition and oxygen supply of the central nervous system to meet the needs of local neurons (Bélanger et al., 2011). Structural and functional damage to the blood–brain barrier and astrocyte dysfunction can lead to NVU dysfunction, resulting in inadequate oxygen and energy metabolism in brain tissue. Nakanishi et al. (2014) ascertained that non-MHE cirrhotic patients exhibited a sharp and repetitive increase in cerebral oxyhemoglobin during task execution, while MHE patients showed a relatively slow increase, indicating poor task responsiveness to cerebral oxygen concentration levels in MHE patients. Sunil et al. (2012) also reported abnormal cerebral blood flow perfusion in multiple brain regions of MHE patients, and that regional cerebral blood flow in some brain regions was related to patients’ cognitive impairment. Near-infrared spectroscopy measurements of NAFLD (Takahashi et al., 2017) and PBC (Duszynski et al., 2020) patients illustrated cerebral hypoxia in these patients. Moreover, significant decreases in glucose, lactate, and tissue oxygen concentration levels were observed in the cortex of BDL-induced MHE rats (Hadjihambi et al., 2022). In conclusion, these findings indicate that there is energy metabolic dysfunction in mild cognitive impairment associated with liver disease.

#### Neurotransmitter metabolic disorders

Impaired glucose metabolism in the brain not only causes damage to brain energy metabolism but also affects neurotransmitter synthesis. In neuronal cells, glucose generated from glycolysis can not only be metabolized into ATP through the TCA cycle, but its product alpha-ketoglutarate (α-KG) can be synthesized into glutamate (Glu) via aspartate transaminase. Glu released into the synaptic cleft can be retaken up by astrocytes and converted into glutamine (Gln) via glutamine synthetase, and then transferred back to neurons where it is reconverted to Glu via glutaminase, a process known as the glutamate-glutamine cycle (Schousboe et al., 2014). Gln generated by astrocytes is also an important pathway for GABA replenishment. Impairment of the glutamate-glutamine cycle is a major cause of hepatic encephalopathy (Limón et al., 2021).

Magnetic resonance spectroscopy (MRS) of ^13^C-labeled metabolic substrates can provide insights into energy and neurotransmitter metabolism in neurons and astrocytes (Sonnay et al., 2017). Zwingmann et al. (2003) used [1-^13^C] glucose labeling to track neurotransmitter metabolism in rats with acute liver failure (ALF) induced by portacaval anastomosis (PCA), from the early stages of hepatic encephalopathy (HE) to the coma stage. The results showed that, compared to the control group, the total Gln and lactate concentrations in the brains of rats in the early stages of HE increased by 2 to 4.5 times, lactate synthesis (^13^C-labeled lactate enrichment) increased by 2.5 times in rats in the coma stage, Gln synthesis (Gln_2_) increased by 10 times in the precoma stage, and Glu synthesis (Glu_4_) decreased in the coma stage, while GABA synthesis did not show significant differences at any stage. Furthermore, Bosoi et al. (2014) used [1-^13^C] glucose and lactate labeling to trace neurotransmitter metabolism in the brains of rats with minimal hepatic encephalopathy (MHE) induced by bile duct ligation (BDL) and found that the *de novo* synthesis of lactate and Gln significantly increased in the brains of MHE rats. These results indicate the presence of neurotransmitter metabolism defects in MHE.

Neuroinflammation can affect the transmission of glutamatergic and GABAergic neurotransmitters (Cabrera-Pastor et al., 2019). Changes in glutamatergic and GABAergic neurotransmission are closely related to cognitive and motor changes in MHE patients (Llansola et al., 2015), and an increase in GABAergic tone in the brain is considered a characteristic of HE. Hassan et al. (2019) used transcranial magnetic stimulation to compare cerebellar inhibition (CBI) in 15 HE patients at different stages with 15 healthy controls. The results showed that CBI was reduced in HE patients at a stimulus interval of 5–7 ms, but the degree of CBI in patients at a stimulus interval of 7 ms was significantly correlated with the severity of HE, indicating an increase in GABAergic neurotransmission in the cerebellum of HE patients and a decrease in GABAergic neurotransmission in the motor cortex. Zöllner et al. (2023) study of 16 healthy controls and 19 MHE patients used edited MRS to measure GABA levels in the right cerebellum, left thalamus, and left motor cortex. The results showed that Gln levels were elevated in all three brain regions in MHE patients, whereas GABA levels were elevated in the cerebellum, and significantly decreased in the motor cortex and highly correlated with the severity of MHE. A clinical study also reported that the GABA-A receptor modulating steroid antagonist golexanolone improved cognition in MHE patients (Montagnese et al., 2021). Overall, these study results imply the important role of changes in the GABAergic system in the cerebellum and motor cortex in MHE.

Besides, neurotransmitter metabolic changes have been reported in patients with liver disease-related mild cognitive impairment. In NASH, rats with cognitive impairment, had decreased dopamine levels in the frontal cortex and the cerebellum and reduced norepinephrine levels in the striatum (Higarza et al., 2019). In patients with chronic hepatitis C cognitive impairment, the choline/creatine ratio in the basal ganglia and posterior cingulate gyrus was significantly increased, while the N-acetylaspartate/creatine ratio was significantly decreased. Cognitive impairment was also significantly negatively correlated with the choline/creatine ratio and positively correlated with the N-acetylaspartate/creatine ratio in the basal ganglia (Abo Hagar et al., 2018). Furthermore, Pflugrad et al. (2019) compared the brain MRS of healthy individuals with LT-related cognitive impairment patients before and after LT and found that after LT, there was an increase in myo-inositol in the thalamus, putamen, and white matter in LT cognitive impairment patients, and a decrease in glutamine/glutamate ratio in the putamen. Patients without cognitive impairment after LT only showed an increase in myo-inositol in the thalamus after LT, and there was no significant difference in brain MRS between transplant-related cognitive impairment patients and those without cognitive impairment.

#### Liver-derived factors

The liver’s condition is closely intertwined with brain function. A study by Seo et al. (2016) revealed that elevated levels of liver enzymes, specifically aspartate aminotransferase (AST) and alanine aminotransferase (ALT), in patients with NAFLD are associated with impaired cognitive performance. Interestingly, in healthy individuals, plasma levels of ALT and AST not only display a significant negative correlation with memory capacity (Kamada et al., 2016), but also exhibit a noteworthy positive correlation with plasma Glu levels. AST and ALT serve as crucial indicators for assessing liver function biochemically, while also acting as vital enzymes involved in hepatic gluconeogenesis and the regulation of tissue glutamate production (Sookoian and Pirola, 2015). ALT facilitates a reversible transamination reaction between alanine and α-ketoglutarate, leading to the formation of pyruvate and glutamate. Conversely, AST catalyzes the reversible reaction between aspartate and α-ketoglutarate, resulting in the production of oxaloacetate and glutamate (Ellinger et al., 2011).

Notably, decreased ALT levels signify reduced liver metabolic activity and have been linked to a higher risk of dementia (Lu et al., 2021; He et al., 2022). Furthermore, a study by Nho et al. (2019)  demonstrated demonstrated that lower serum ALT levels and an elevated AST/ALT ratio in individuals with Alzheimer’s disease (AD) are associated with poorer cognitive abilities, reduced brain glucose metabolism, and the deposition of amyloid-beta (Aβ). These findings suggest that reduced ALT levels may contribute to decreased pyruvate levels, leading to disrupted energy balance, compromised glutamate metabolism, and impaired synaptic transmission in the central nervous system. However, it’s important to note that Ferri et al. (2022) only observed lower serum ALT levels and higher AST/ALT ratios in elderly male dementia patients compared to cognitively normal individuals, with no significant differences observed in elderly females. In contrast, a study by Yokokawa et al. (2022) found a significant correlation between serum levels of brain-derived neurotrophic factor (BDNF) and liver enzymes in middle-aged and elderly females, along with a negative correlation between BDNF and liver fibrosis measured by the Fibrosis-4 (FIB-4) index. Although cognitive function was not assessed in this particular study, previous research has established a link between liver fibrosis and an increased risk of dementia (Weinstein et al., 2019; Solfrizzi et al., 2020; Jeong et al., 2022). Moreover, serum BDNF has been identified as a biomarker not only for cognitive function in elderly females (Komulainen et al., 2008) but also for the decline in cognitive abilities associated with mild cognitive impairment and dementia (Nikolac Perkovic et al., 2023). In addition, a study by Chen et al. (2020) reported that short-term exposure to bilirubin can induce AD-like brain pathology. In summary, while gender differences exist, liver enzymes play a significant role in the underlying mechanisms of dementia.

The liver, as the largest organ responsible for metabolite clearance in the bloodstream, plays a critical role in the peripheral clearance of plasma Aβ. Peripheral clearance of Aβ by the liver potentially facilitates the removal of Aβ from the brain, thus contributing to the treatment of AD (Liu et al., 2015). Impairment in liver function leading to reduced peripheral Aβ clearance is a significant factor in the accumulation of Aβ in the brain and the development of AD (Bassendine et al., 2020). A study by Cheng et al. (2023) revealed elevated plasma Aβ levels in patients with liver cirrhosis, and chronic impairment in hepatic Aβ clearance was found to increase brain Aβ deposition in APP/PS1 transgenic mice, resulting in neurodegeneration and cognitive deficits. These findings suggest that a decrease in hepatic Aβ clearance rate may be a potential factor in AD development. Additionally, Li et al. (2013) reported postoperative cognitive dysfunction in 44% of patients who underwent liver transplantation, which was associated with an increase in postoperative serum biomarkers of dementia, including Aβ, within 24 h after surgery. Moreover, NAFLD exacerbates Aβ plaque accumulation in transgenic AD mice, promoting the progression of AD (Kim et al., 2016). Furthermore, the clearance of peripheral Aβ relies on the transportation of circulating proteins such as albumin and apolipoprotein E, highlighting the significance of lipid metabolism disorders in the liver as contributing factors to Aβ accumulation and cognitive impairment (Cheng et al., 2020).

Furthermore, certain liver-derived factors have the potential to regulate cognition. For instance, Horowitz et al. (2020) reported that an elevated concentration of liver-derived glycosylphosphatidylinositol (GPI)-specific phospholipase D1 in the plasma of mice after exercise can improve hippocampal function and alleviate cognitive impairment in elderly mice. Yan et al. (2022) found that aerobic exercise increases the synthesis of liver methyl donors, leading to enhanced brain RNA N6-methyladenosine levels and exhibiting anti-anxiety effects. These research findings collectively suggest that liver-derived factors play a significant role in mild cognitive impairment associated with liver disease.

### Research progress on magnetic resonance imaging of mild cognitive impairment in liver disease

Recently, the development of Magnetic Resonance Spectroscopy (MRS) and Magnetic Resonance Imaging (MRI) techniques has provided non-invasive detection methods for exploring changes in brain structure and function in cognitive impairment (Chavarria and Cordoba, 2015). MRS detects the proton signals of metabolites and can distinguish the concentration of metabolites such as lactate, Glu, Gln, and GABA in the brain. Further, by adding substrates with special radioactive labeling such as ^13^C, it is possible to track the metabolic processes of compounds, and MRS related to neurotransmitter metabolism has been discussed in previous reviews. Brain MRI mainly images water proton signals in brain tissue. Conventional MRI images include T1 and T2-weighted images, which can provide information on the anatomy, metabolism, and water content of the brain. Moreover, functional Magnetic Resonance Imaging (fMRI) can assess the function of brain regions based on changes in blood oxygen signals generated by neuronal activity in brain functional areas and establish brain functional networks. Numerous studies have shown that the progress in mechanism research on mild cognitive impairment related to liver disease is closely related to the application of magnetic resonance brain imaging technology (see Table 1).

**Table 1:** Advances in magnetic resonance imaging for mild cognitive impairment in liver disease.

Author	Study	Imaging methods	Relationship between imaging and cognitive impairment
Marciniewicz et al. (2019)	Before and after HCV antiviral therapy	3D-FSPGR and T2-FLAIR	After antiviral treatment, cognitive improvement is observed in HCV patients, and there is a decrease in the volume of certain brain regions.
Zhang et al. (2020)	HCV	fMRI，Function Connectivity (FC)	FC changes not significantly correlated with patient cognitive impairment
Cheng et al. (2021a) and Cheng et al. (2021b)	Impaired cognition related to LT in patients with OHE or non-OHE cirrhosis	Resting-fMRI，FC	Restoration of normal FC in non-OHE cirrhotic patients after LT, while partial abnormal FC still persists in OHE patients.
Xu et al. (2023)	NAFLD	Resting-fMRI，Amplitude of Low Frequency Fluctuation (ALFF)	The ALFF value of the cingulate gyrus and the FC strength between the left middle temporal gyrus and the right inferior frontal gyrus are correlated with the decline of cognitive ability in patients.
Mosher et al. (2017)	PBC	Resting-fMRI，FC	The weakened FC between the patient’s amygdala and the right inferior occipital gyrus, left inferior frontal gyrus, and posterior cingulate cortex is associated with lower cognitive test scores.
Reichardt et al. (2022)	HCV, Autoimmune Hepatitis, and PBC patient	MRS and MRI	MRI/MRS changes are not correlated with patient cognitive impairment.
Lin et al. (2019), Cai et al. (2022), Zhan et al. (2019), and Guo et al. (2022)	MHE	Resting-fMRI	FC changes are associated with cognitive scores in MHE patients with cirrhosis.
Li et al. (2019) and Sato et al. (2019)	MHE	Diffusion kurtosis imaging (DKI) and Diffusion tensor imaging (DTI)	Changes in DKI are associated with cognitive impairment in patients with MHE.
Nho et al. (2019)	AD	MRI	ALT level and ALT/AST ratio are associated to glucose metabolism and cortical thickness
Li et al. (2022)	AD	MRI	The AST/ALT ratio is negatively correlated with the volume of the right hippocampus.

In the healthy population, there is a significant relationship between serum liver function markers and brain structure and function. Chen et al. (2021) discovered a positive correlation between serum proteins and gray matter volume (GMV) in the parahippocampal gyrus and amygdala. The levels of globulin and the albumin/globulin ratio were associated with GMV in the olfactory cortex and parahippocampal gyrus. Higher bilirubin levels were linked to increased regional homogeneity (ReHo) in the precentral gyrus, middle cingulate gyrus, and inferior frontal gyrus, while ReHo decreased in the caudate nucleus. Furthermore, elevated ALT levels were found to be correlated with increased cerebral blood flow (CBF) in the superior frontal gyrus and decreased CBF in the occipital gyrus, angular gyrus, precentral gyrus, and middle temporal gyrus.

In MHE patients, T1-weighted images mainly show high signal intensity in the globus pallidus and thalamic edema. Taylor-Robinson et al. (1995) compared the MRI findings of MHE patients, OHE patients, and patients with chronic liver cirrhosis but without cognitive impairment, and found that the signal intensity of the globus pallidus was significantly higher in patients with cognitive impairment than in those without, and that the T1 signal intensity of the globus pallidus was correlated with blood ammonia levels. However, this increased magnetic resonance signal intensity in the globus pallidus is believed to be caused by manganese deposition, as the T1 high signal is related to blood manganese concentration levels in patients with liver cirrhosis (Taylor-Robinson et al., 1995), and the manganese content in the globus pallidus was also shown to be significantly increased in HE patients on autopsy (Butterworth et al., 1995). Lin et al. (2022) also compared brain T1-weighted images of healthy individuals and patients with hepatitis B-related cirrhosis and illustrated that patients with cirrhosis had significant thalamic swelling even before the onset of MHE, which further extended to bilateral basal ganglia and the cortex. Cerebellar swelling occurred during the MHE stage, and thalamic swelling was significantly negatively correlated with cognitive ability in MHE patients. Winterdahl et al. (2019) likewise found increased water content in the brain white matter and thalamic edema in patients with mild HE, that can lead to damage to the basal ganglia-thalamus-cortex loop. Brain edema and increased water content were observed in MHE patients on T2-weighted images. Diffusion-weighted imaging (DWI) can be used to evaluate the diffusion ability of water molecules in tissues using the apparent diffusion coefficient (ADC). Sugimoto et al. (2008) demonstrated that the brain ADC value increased in MHE patients with liver cirrhosis, indicating significant brain edema in MHE patients, and that the ADC value of the frontal and parietal lobes could predict the progression of HE.

The high signal intensity of the globus pallidus in liver disease patients can recover within 5 months after liver transplantation (LT) (Long et al., 2009). Compared with LT patients without cognitive impairment, LT patients with cognitive impairment showed high signal intensity in the bilateral insular cortex and cingulate gyrus and extensive brain edema. MRI findings in non-alcoholic fatty liver disease (NAFLD) patients indicate white matter lesions (Petta et al., 2016) and reduced brain volume (Weinstein et al., 2018), and the relationship between decrease in brain volume and cognitive impairment (Filipović et al., 2018). In chronic hepatitis C, Thames et al. (2015) found that fractional anisotropy scores in the striatum of HCV patients with cognitive impairment increased as well as the mean diffusion coefficients in the cingulum and external capsule, and that the diffusion coefficient in the superior longitudinal fasciculus was significantly correlated with patient cognitive performance. Prell et al. (2019) also reported that compared with the control group, HCV patients with cognitive impairment had gray matter atrophy in the bilateral insular cortex and thalamus, increased gray matter volume in the cerebellum, and that gray matter atrophy in the left amygdala and left parahippocampal area worsened during the 7-year disease progression.

Additionally, changes in functional connectivity (FC) of the brain are also important causes of mild cognitive impairment (MCI) related to liver disease. Ye et al. (2020) compared interhemispheric functional connectivity and the corpus callosum volume between healthy controls, patients with minimal hepatic encephalopathy (MHE), and those with cirrhosis without MHE, and reported that patients with MHE showed corpus callosum degeneration and interhemispheric connectivity disorders. At the global level of the brain, Gou et al. (2020) determined that the small-worldness of the brain structural network was significantly reduced in patients with MHE, and the network integration and module separation were decreased. Brain functional connectivity significantly improved after LT treatment in patients with HE (Ahluwalia et al., 2016). However, Cheng et al. (2018) showed that after LT treatment, most of the abnormal functional connectivity strength in patients without HE returned to normal levels, but HE patients still retained most of the abnormal functional connectivity strength, including brain areas related to advanced cognition such as the frontal and parietal lobes. This suggests that the recovery of brain functional connectivity after LT is influenced by the history of HE before transplantation. In summary, magnetic resonance imaging can provide a non-invasive method for studying brain metabolism, structure, and function in patients with liver disease-related MCI, which is helpful for exploring the mechanisms related to cognitive impairment and for diagnosing and treating liver disease-related MCI in clinical practice.

## Conclusion

Liver diseases such as MHE, NAFLD, cholestatic liver disease, and mild cognitive impairment in liver transplant recipients are very common. Cognitive impairment not only seriously reduces the quality of life of patients but can also progress to overt hepatic encephalopathy, leading to poor prognosis for patients. This review summarized the mechanisms underlying mild cognitive impairment in liver disease, including high blood ammonia, neuroinflammation, brain energy metabolic disorders, and neurotransmitter metabolic disorders, liver-derived factors, as well as the emerging research progress in magnetic resonance imaging of the brain, in order to provide ideas for the prevention and treatment of mild cognitive impairment in liver disease.

## Author contributions

All authors listed have made a substantial, direct, and intellectual contribution to the work and approved it for publication.

## Funding

This work was supported by the National Natural Science Foundation of China (Nos. 82070302 and 81873467).

## Conflict of interest

The authors declare that the research was conducted in the absence of any commercial or financial relationships that could be construed as a potential conflict of interest.

## Publisher’s note

All claims expressed in this article are solely those of the authors and do not necessarily represent those of their affiliated organizations, or those of the publisher, the editors and the reviewers. Any product that may be evaluated in this article, or claim that may be made by its manufacturer, is not guaranteed or endorsed by the publisher.
